# Researching Men's Violence Against Women as Feminist Women Researchers: The Tensions We Face

**DOI:** 10.1177/10778012221134823

**Published:** 2022-11-03

**Authors:** Sandi Dheensa, Karen Morgan, Beverly Love, Helen Cramer

**Affiliations:** 1 Domestic Violence and Abuse Health Research Group, Centre for Academic Primary Care, Population Health Sciences, 1980 University of Bristol, Bristol, UK; 2 National Addiction Centre, Institute of Psychiatry, Psychology & Neuroscience, 4616 King's College London, London, UK

**Keywords:** domestic violence, perpetrators, interviews

## Abstract

Qualitative and feminist researchers aim to build rapport, show empathy, be non-judgemental, and equalise power imbalances. A crucial challenge researchers face is how to navigate and balance competing aims and values when interacting with and interviewing participants who have perpetrated intimate partner violence and abuse towards women. In this article, four female researchers evaluating perpetrator programmes for abusive men use reflexive analysis to identify the tensions encountered in such research. We outline how these tensions affected us and the data produced, and end with recommendations, which we hope will help prepare researchers, particularly women, for conducting interviews with violent/abusive men.

## Introduction

Intimate partner violence and abuse (IPVA) is a form of domestic violence and abuse and includes physical, sexual, psychological, financial, and other forms. Physical and sexual IPVA affects a third of women worldwide (Sardinha & García-Moreno, 2021). In England and Wales, between 2010 and 2020, an average of 80 women a year were killed by a partner or ex-partner (Office for National Statistics, 2021). Although men can be victims and women can be perpetrators, IPVA is most commonly perpetrated by men against women. Hamberger and Larsen's (2015) review of comparative studies about IPVA among heterosexual couples showed that when women use physical violence against men, it is often in response to violence that men have used against them. Regarding psychological and/or emotional abuse, men, unlike women, tend to use tactics that threaten women's lives and constrain their autonomy. Men are also the main perpetrators of sexual abuse. Compared with men, women are more highly victimised, injured, and fearful (Hamberger & Larsen, 2015). Stark (2012) argues that men use IPVA to secure male privilege and domination and that political power is “created when men as a group use their oppressive tactics to reinforce persistent sexual inequalities in the larger society” (p. 8).

Interview-based research with male perpetrators has explored the way they talk about their use of violence and abuse against women and has found that they use various narrative and discursive strategies to present themselves as generally nonviolent and rational, such as minimising, denying, and justifying their actions (e.g., Adams et al., 1995; Anderson & Umberson, 2001). As Hearn (1998) argues, men's use of these discursive strategies presents a methodological difficulty in conducting the research, in that they can be barriers to collecting rich descriptive data, but they are also features of men's use of violence and abuse—arguably findings in and of themselves.

Although the body of interview-based research with male perpetrators of violence and abuse against women is growing, only a small body of literature reflects on the process of doing such research, and the tensions and challenges the process produces. In this article, we—four feminist women researchers, who use primarily qualitative and feminist-inspired research methods—explore how our approaches to research unfolded with such men. Unlike the research reflected upon in the existing literature, our research was mixed methods, comprising semi-structured qualitative interviews and structured interviews: although these produced quantitative data, we drew on our qualitative research skills to administer them.

We now outline the meaning of ‘good interviewing’ and what feminist-inspired interviewing emphasises, before reviewing the small body of literature about research with men who have used violence and abuse against women.

### Good Interviewing Practice

Qualitative interviewing aims to foreground and explore participants’ thoughts, views, feelings, experiences, and “framework of meanings” (Britten, 1995, p. 251). Although the researcher predetermines the issues to be explored, they avoid imposing their own “structures and assumptions as far as possible” (Britten, 1995, p. 251). Scholarship on what constitutes good qualitative interviewing emphasises that interviewers should pose questions and use probes that are non-directive, neutral, and clear; should not interrupt or disagree with interviewees; and should be sensitive to participants’ choice of language and use of concepts (Boonzaier, 2014; Britten, 1995; Patton, 1987). Researchers should also establish and maintain rapport. Rapport is defined in varying ways: the degree of comfort in interviewer-participant interactions (Morgan & Guevara, 2008), interviewers’ communication of empathy and understanding without judgment (Patton, 2015, p. 458), and interviewers’ signalling of solidarity with the participant (Prior, 2017). Along with a relationship of trust, acceptance, empathy, and mutual respect with the participant (Bluff, 2005; Rapport, 2005; Taylor, 2005), rapport is thought to make rich accounts and participant reflections on the phenomenon of interest more likely. These same skills are relevant for structured (quantitative) interviews.

Researchers have recognised that interviewers hold power over participants through being in a position of authority and being able to set the agenda of the interview beforehand. Some researchers have argued that this interviewer-participant power imbalance should be attenuated: interviewers must be ready to surrender control to participants to enable them to tell their own stories in their own ways (Taylor, 2005). Researchers whose approach is inspired by feminist methodology put particular emphasis on attenuating power imbalances between the ‘expert’ researcher and the ‘researched’ when conducting research with women (Doucet & Mauthner, 2012). Writing in the 1980s and 1990s, feminist researchers argued that although certain groups of women have more power than others, women are always relatively powerless in a patriarchal society, and traditional social sciences have tended, at least in the past, to be imbued with patriarchy. As a result, we as women have been more likely than men to have our experiences discredited, and we have had the power of naming our own experiences taken from us: our experiences have instead been named by men (Finch, 1984; Oakley, 1981; Stanley & Wise, 1993). Feminist methodology, which can be qualitative or quantitative, attempts to redress this issue. It focuses on gender, power, and inequality and feminist interviewers may be especially concerned with ethical conduct, feel they have an additional duty to raise consciousness around oppression, and be keen to include reflexivity in research practices (DeShong, 2013; Harding, 1987; Hesse-Biber, 2011; Olsen, 2011; Stanley & Wise, 2013; Taylor, 2005).

Reflexivity entails researchers being aware of the multiple intersecting positions we hold (e.g., according to gender, race, class) in relation to both the research questions and participants, as well as being aware of the emotions, motivations, assumptions, biases, and values we bring to the research process (Steedman, 1991). These factors, along with the interviewer-participant interaction, and the wider social context in which the research is conducted, affect and shape the data that is being produced. Being reflexive is a way to acknowledge that different realities and forms of knowledge exist, that varied factors impinge on knowledge production, and that knowledge cannot be separated from the knower (Ardener, 2006; Stanley & Wise, 2013; Steedman, 1991). Reflexive examination is therefore a way to help dismantle ‘hierarchies of knowledge’ i.e., where researchers are seen as the ‘expert knowers’ who know more and know better (Oakley, 1998; Stanley & Wise, 2013). It also makes explicit the power dynamics inherent in the interviewer-participant relationship. Intersectionality-informed interview approaches (Crenshaw, 1991; Hunting, 2014) encourage researchers to explore more explicitly how social categories can interact to increase the subordination and oppression faced by, for example, women of colour.

Another aspect of interviewing that has been emphasised by feminist researchers, and taken up by IPVA researchers interviewing women (e.g., Morgan, 2014), is reciprocity, whereby researchers share personal views or stories of personal experiences with participants. This sharing helps the researcher to break down the voyeuristic and exploitative nature of research, build trust and acknowledge commonalities between themselves and the participant, and validate the participants’ experiences (Gadd, 2004; Oakley, 1981). The approach replaces the ‘faked friendship’ aspect of rapport that is built with the primary aim of ‘obtaining’ research data (Morgan, 2014).

### Challenges Faced in Research with Male Perpetrators of Violence Against Women

As Boonzaier (2014) argues, IPVA is a morally reprehensible behaviour and conducting interviews with male perpetrators presents particular challenges for researchers, especially when the researcher is a woman and a feminist. A crucial challenge researchers face is how to navigate and balance competing aims and values when interacting with and interviewing participants who have perpetrated IPVA towards women. Although interviewers usually aim to create a comfortable and non-judgmental space, doing so may be difficult if a female researcher does not feel comfortable herself because the participant is being offensive, upsetting, or threatening (Gailey & Prohaska, 2011). The notion that research ought to be empowering to participants is also complicated when conducting research with violent men (Chung & Zannettino, 2006) given that men arguably use IPVA to secure power over women (Stark, 2012). However, Presser (2005) argues that we, as feminist researchers, should do more to explore power relations in research with such men and “expose the marginalisation of those violent male subjects who speak to us” (p.2068) e.g., in relation to race and class.

While female researchers may face certain challenges when interviewing abusive men, so might male researchers. For example, interviewees may be more likely to make attempts to get a male interviewer on their side and build camaraderie with them. They might denigrate women and expect male interviewers to join in (Gottzén, 2013; Hearn, 1998). Hearn (1998) writes that managing these tensions involves balancing “attention, listening, empathy [along with] critical distance and critical awareness” (p. 55).

Few researchers have reflected on the challenges women face in conducting interview-based research with and about men who have used violence against women (Gailey & Prohaska, 2011; Huysamen, 2016; Presser, 2005) or IPVA specifically (Boonzaier, 2014; DeShong, 2013; Hydén, 2014). We aim to add to this literature by exploring the tensions we experienced in working on two pilot/feasibility randomised controlled trials (RCTs) of ‘domestic violence perpetrator programmes’, ADVANCE (Dheensa et al., 2021; Gilchrist et al., 2021) and REPROVIDE, for men who had used IPVA against women. We experienced these tensions through our positions and roles as (qualitative) researchers working within the constraints of our research designs, but also as women and as feminists, wanting our research to make a difference in the lives of victim-survivors and their families. We found that these different positions, roles, and responsibilities pulled us in different directions. In this article, we explore how we navigated these tensions, how they affected the data produced, and how they compared with the tensions explored in the existing literature.

We reflect in this article on our use of semi-structured qualitative interviews and structured interviews (which produced quantitative data). These structured interviews were made up of a battery of measures, and we read their items aloud to men. Participants often elaborated on their answers, meaning we drew on qualitative research skills in their administration. Our article in this way is unique, and we highlight the particular tensions that emerged from doing qualitative research within trials, where the research aims to generate evidence about specific questions. By presenting our reflections overall, we hope that we can also shed light on the possibilities within feminist-inspired interview-based research, and develop these research practices (Chung & Zannettino, 2006).

## Method

Before outlining the method that we adopted for the analysis presented in this article, we provide some background to the data collection and the main intervention elements of the two pilot/feasibility RCTs in question. The research activities upon which we reflect took place mainly between 2018 and 2019. SD and BL were the researchers on ADVANCE, which was specifically for abusive men in treatment for substance or alcohol use. They recruited men from substance use treatment services by approaching them in waiting rooms or at the end of treatment groups. KM and HC were the researchers on REPROVIDE: abusive men could self-refer or be referred by any professional, e.g., from social care or healthcare. In ADVANCE we used the Adapted Abusive Behaviour Index (Postmus et al., 2016) to screen men for eligibility: men were required to have used just one behaviour in the past 12 months to be eligible, based on the assumption that some men will deny and minimise behaviour. REPROVIDE screened men against basic eligibility criteria before doing a fuller joint assessment for suitability and motivation to change with IPVA specialists. In both studies, eligible men were invited to complete structured baseline interviews, in which we administered multiple measures (e.g., on mental health, post-traumatic stress disorder, and adverse childhood experiences). After the baseline, we randomly allocated men to the intervention group or control group (which did not receive an intervention). Both ADVANCE and REPROVIDE comprised one-to-one preparatory session(s) followed by weekly group sessions (ADVANCE 12 weeks, REPROVIDE 23 weeks). We interacted with men throughout the intervention period, e.g., by sending text reminders and collecting evaluation forms from them. Regarding follow-ups, REPROVIDE researchers followed men up at three monthly intervals, while ADVANCE researchers administered a structured and semi-structured qualitative interview at the end of the intervention (Gilchrist et al., 2021). Interviews were audio-recorded and transcribed. In both studies, female partners and ex-partners were contacted and offered (or signposted to) specialist support and the chance to be interviewed.

During ADVANCE and REPROVIDE, we met regularly to discuss emergent findings and reflect on the research process. SD, KM, and HC also discussed their experiences in a ‘perpetrator reading group’. Reading the article by Boonzaier (2014) inspired us to look back over interview transcripts and recruitment fieldnotes and reflect on moments in which we felt discomfort, dissonance, or tension. Through discussion and writing, we conducted a secondary discursive and reflexive analysis of these transcript segments and fieldnotes, which we present here. In presenting quotations from interviews, we take heed of Presser (2005) who argues that feminist researchers studying men's violence should write themselves, i.e., their interjections and reflections, into their work. We do not report on interviews with female partners and ex-partners here.

## Findings

We present six overlapping themes representing tensions that emerged from our analyses. Within each, we provide quotations from qualitative interviews and extracts from fieldnotes to illustrate where and how the tensions manifested. Men's names are pseudonymised. That our interviews and reflections were gathered in the context of evaluating perpetrator programmes is relevant to all tensions: to an extent, we were framing men as perpetrators of abuse in need of behaviour change, and men accepted this framing to varying degrees. All tensions were amplified when colleagues had interviewed men's female partners, and in all cases, we interviewed men while keeping these partners in mind.

### Using the Term ‘Domestic Violence’ or Careful Euphemisms

A tension that arose when recruiting men for ADVANCE and REPROVIDE was around what sort of language to use when talking with the men about the programmes. Should we be direct, calling ‘domestic violence’ by its name? Or should we use careful language to avoid stigma and build trust, perhaps even mirroring men's own use of language (Britten, 1995)? We were concerned that some men might have felt the programmes were irrelevant to them upon hearing the term ‘domestic violence’, including because they might not have recognised, or might have been refusing to recognise, their behaviours as abusive and violent. As Hearn (1998) points out, “when talking to men as the doers of violence, it is far from clear what violence is to be taken to mean.” (p. 66). Men not recognising their violence and abuse is especially common when the forms are non-physical (Kelly & Westmarland, 2016). Other men might have felt angry and stigmatised by the terms. Thus, using such language might have led to fewer men taking part overall, which would have meant a missed opportunity to generate robust evidence for an intervention that could be valuable for women's safety. Building rapport and having a non-judgemental approach was as important at this stage as it was in interviews. We did not want to deter men from talking openly during screening and interviews, and in turn, produce data that were less complete and rich (Bluff, 2005; Rapport, 2005; Taylor, 2005).

For these reasons, during recruitment, screening, and, in some cases, in the structured baseline interviews, we steered away from the term ‘perpetrator’. Our decision aligned with advice from Iwi and Newman (2015). In guidance for practitioners (e.g., social workers), they recommend that in the initial stages of working with men who use abusive behaviours, one should “speak in language that fits [the] person's current experience” because men “are unlikely to relate to being called a domestic violence perpetrator just now” (p. 31). Similarly, Tu and Penti (2020), writing for physicians, argue that “while people who perpetrate IPV need to be held accountable…labels such as perpetrator, abuser, batterer, rapist, may alienate those who could be open” to discussing their behaviours (p. 809). To an extent, SD and BL avoided the term ‘domestic violence’ as well as the term ‘perpetrator’ in ADVANCE, while in REPROVIDE, HC and KM used this term more frequently. The REPROVIDE researchers wanted men to be clear about the programme's focus and needed men to admit to some sort of abuse at the start of the programme and be motivated to change that behaviour. Men in REPROVIDE had self-referred or been referred to the programme by social services, so were perhaps more ready to hear direct language than men in ADVANCE, who were approached in services by researchers and not always aware beforehand of the nature of the research. On ADVANCE, we also wondered whether using these terms would have turned our discussions and structured interviews into interventions in themselves, which could have made it more difficult to identify aspects of the programme that led to change. Instead of using these direct terms, we sometimes used euphemisms like ‘relationship conflicts’ and described abusive and violent acts (e.g., controlling money, following the partner, physical altercations).

These alternative phrases and euphemisms could only take us so far. In ADVANCE, one of the baseline measures contained the word ‘violence’ in every item. And in some cases, men's follow-up interviews vindicated our assumptions that words and phrases like these might stigmatise and alienate them. In his end-of-study qualitative interview, Adrian, who had disclosed using non-physical abuse and was in the control group so did not receive the ADVANCE intervention, told SD, “I was really uncomfortable with that [the word violence]…I’m thinking, ‘what am I letting myself in for?’…People would think I was physically violent”. SD had told Adrian while administering the baseline measure that violence was broader than just physical violence, but in his end-of-study qualitative interview he said, “It's not obvious that violence means emotional violence, that's a very sophisticated interpretation of the word violence, and I don't like that either.” In his end-of-study qualitative interview with SD, Jeff similarly reflected on hearing the word ‘violence’ in the baseline measures: “I thought, ‘Well, I’ve not ever hit anyone’ …those words can be quite powerful and almost a bit confusing…[and] difficult.” Like Adrian, he had perpetrated non-physical abuse, but he received the ADVANCE intervention. He said he preferred and identified more with the term ‘intimate partner abuse’, which felt to him more expansive and relevant to the types of abuse he used, but he came to identify with this term only through the ADVANCE programme.

In other cases, our worry that terms like ‘domestic violence’ would cause men to become alienated and stigmatised was unnecessary. Billy was one example of such a case. He had been in the ADVANCE control group, so unlike Jeff, had not grown accustomed to hearing his behaviour described as abusive or violent. And so, in the end-of-study interview, SD found herself continuing to avoid these terms where possible. However, towards the end of the interview, Billy said, “Basically, it was about domestic violence, really.” With this, he revealed that despite SD discursively dancing around the topic, he knew exactly what her euphemisms implied. He continued, “I’ve been through domestic violence myself when I was younger. I didn't ‘do time’ for it, I did probation. So, I know exactly what it's all about because I had to do that domestic violence course.” Had SD used the phrase ‘domestic violence’ in their first meeting, some interesting and useful discussion might have emerged. However, as described in a later section, Billy came into that first meeting feeling angry. Indeed, sometimes our choices about language were based on how the men made us feel—how comfortable and safe we felt as researchers—as well as on needing the men to feel open to wanting to take part.

### Using Rapport and Reciprocity Without Collusion

Although we were interviewing men on account of behaviour that is considered morally reprehensible, we still strove to build rapport with them. We wanted to engender trust to encourage disclosure and discussion (Bluff, 2005; Rapport, 2005; Taylor, 2005). We even identified a few examples where SD and HC employed reciprocity, i.e., mutual disclosure. The tension here was in not going so far with rapport and reciprocity as to feel that we were colluding with men. HC's qualitative interview with Charles, who had completed the REPROVIDE programme and had just called himself ‘an angry person’, illustrates an example of this tension:

HC: It sounds like you wouldn't describe yourself as an angry person now. Or much less so?

Charles: 80% less. Everybody has still got it in them.

HC: Yes, my partner says when I’m driving, that's my sore point.

Charles: I’ve had 1 or 2 slip-ups when driving because driving is one of my things.

HC: There are idiots on the road.

Charles: When people drive a car badly, they can kill you…It's just like giving some idiot a gun…sometimes I have a little outburst.

Charles presented “everybody has still got it in them” as a factual statement, in a way normalising his anger. HC replied “yes”, acknowledging that his statement was correct and then disclosed, as a way of showing empathy and understanding, that she is sometimes an angry driver. HC added, “there are idiots on the road” in response to Charles saying he has had “slip-ups” while driving. The issue here is that Charles may have interpreted HC's interjections to mean that she felt his “slip-ups” and “little outbursts” (phrases that hint at minimisation) were reasonable. Boonzaier (2014) has highlighted a similar finding from her own data. While reflecting on her transcripts, she found that she had responded to a man's minimising of violence, “I mean it's like, I trashed my room, it's like…”, with “yes, yes, what does it have to do with her?” (p. 240). She writes that she was dismayed at her own response and reflects that she may have “been performing the role of the ‘good interviewer’ through a concern with gaining rapport and through the use of encouraging language to facilitate trust” (p. 241). The same issue can be observed in HC's extract. Boonzaier continued, “The concern [is] whether my interjection…was interpreted…as collusion [of his violence] as harmless to women.” (p. 241). DeShong (2013) has pointed out that even by using subtler rapport-building gestures like nodding and interjections like ‘I understand’, we run the risk of unwittingly endorsing men's violence. Like us, she found herself “walking a fine line” between getting men to talk about their violence while not conveying support for their “misogynist practices” (p. 13). We too felt that ‘appropriate rapport’ was a fine line: on one side was men interpreting us as vindicating them of their actions. On the other was men seeing us as detached, which might have deterred them from continuing to participate and engage in open discussion.

Another example of reciprocity and mutual disclosure came from SD's interview with Dylan, a man of colour. Dylan mentioned that there was friction with his new partner (towards whom he alleged no IPVA) over her reluctance to introduce him to her parents. His explanation for her reluctance consisted of just four words: “she's a white girl.” SD, a woman of colour, understood that Dylan meant this to mean ‘her parents are racist’ and that by using this ‘shorthand’, he was positioning her as someone with whom he might share an experience of oppression and as someone who might understand the subtext behind the four words. DeShong (2013) found that female participants used short-hand phrases akin to ‘you know what men are like’, which accorded her an ‘insider status’, based on their assumptions about her sexuality and gendered experience. But in SD's example, it was a man according her this insider status. SD told Dylan about something similar she had experienced in response, feeling that self-disclosure was a natural conversational response that would encourage the discussion to flow. But she found it emotionally jarring to suddenly go from relating to Dylan by virtue of his violence against women to relating to him as a person of colour with whom she shared experiences of racism. Our intersectional positions, and our connections to the ways in which men were genuinely disempowered in society, made the fine line between rapport and collusion even more difficult to traverse.

### Whether to Challenge and Call Out Men's Abusive Behaviours

Linking closely to the problem of whether to use terms like domestic violence, a common issue we faced was the wish to establish a context in which men felt comfortable to speak, in tension with the moral obligation to challenge or call out men's problematic views and behaviours. This tension was underpinned by our roles as interviewers who do not judge or interrupt what participants say and allow them to share their thoughts, views, feelings, and experiences (Patton, 2015), and our positions as women and as feminists who are the victims of men's misogynistic views and who want to challenge patriarchy. During our research, these roles and positions were, in some ways, at odds. Others have also experienced this tension. In her reflexive analysis of interviews with men who pay for sex, Huysamen (2016) reflects, “there were…many opportunities where I could have challenged a participant's assumptions and statements about gender, sexuality and race. Instead, I had nodded my head or given an understanding ‘mmm’ to sentiments I sometimes wholeheartedly disagreed with” (pp. 24–25). Gailey and Prohaska (2011) warn that by not expressing shock or outrage in response to what men say, researchers run the risk of reinforcing interviewees’ views. In IPVA research, taken to its extreme, this might lead men to go home to their partners feeling vindicated. We found numerous examples where we did not challenge men, including KM's interview with Greg:

Greg: Recently when we’ve been arguing, she’ll say something to me. I said, “Right, I haven't just said that at all. What I think I’m going to do is take myself out of the room because we’re not listening to each other. When you want to talk to me and when we listen to each other, then I’ll be happy to do that…At the moment, I’m answering your questions and you’re interpreting them in a completely different way to what I’m saying. Until we’re calmer our brains aren't going to allow us to make those connections.” I don't know if that pisses her off even more because I’m being objective in an emotional situation.

KM: It's difficult, isn't it?

Although KM's interjection “it's difficult, isn't it” could be read simply as a neutral response to Greg's narrative, on re-reading this transcript, KM felt she ought to have challenged Greg's viewpoint of being “objective in an emotional situation” as a harmful stereotype of women as emotional, hysterical, and irrational. What's more, KM realised that her response could arguably be interpreted as collusion: she acknowledged that Greg was in a difficult situation because of his partner's actions and that he was trying to find a way of handling the situation without “piss[ing] her off even more”. At another point in the interview, Greg said he had hit his partner as he “wanted her to be quiet”:

Greg: I actually never wanted to hurt her actually…I wanted her to be quiet. Actually, I don't know if that's true either. I just wanted the argument to stop. I don't want drama in my life. I want there just to be calm.

KM: No, I think a lot of men feel the same way. I think that's why a lot of people have come to us, because they don't want the drama.

In the interview itself, KM felt uneasy at what he had said about wanting “her to be quiet” because it sounded eerily similar to the defence some men give in court after killing their female partners. At the same time, KM felt that because she was not a therapist or clinician, she could not probe what he had said further. Instead, although not consciously, KM drew on her interview skills in adopting the same words he had used (“the drama”), as well as the description of “a lot of men/people” to reassure and empathise. Her interjection is an example of something Huysamen (2016) has observed: that “women frequently function as facilitators of men's narratives – we seldom interrupt (let alone challenge or critique) men's speech. Rather we are constructed as empathetic listeners who are there to help along men's talk” (p. 24). Indeed, earlier in the interview, KM felt that Greg was despairing, and she remembers almost trying to soothe him.

SD also encountered a situation with Harold in which she felt uncomfortable about whether to challenge his narrative:

SD's FIELDNOTE: When screening Harold for eligibility, he disclosed an alleged ‘one-off incident’ of violence: he had grabbed his wife by the throat and shaken her. I asked for his wife's phone number and saw that her number was saved in his phone as ‘Bitch’. Two weeks later, during the structured baseline interview, Harold elaborated on the ‘incident’, claiming that he’d ‘lost it’ in a ‘moment of rage’ because she was ‘running a brothel’ from his house. He described that after the ‘incident’, he burst into tears, went into the garden, and his wife called the police. When the police officer arrived, he apparently looked around at the chaos Harold's wife had caused, turned to Harold, and said ‘mate, get a divorce.’ On telling me this story, Harold seemingly started to sob, immediately covering his face (including his eyes) with a handkerchief.

If a participant started to cry in most other interviews, offering to stop and continue at another time would be the obvious thing to do. However, SD wondered whether to offer this choice, given that Harold's actions (adopting the victim role, blaming the woman, minimising actions as one-off incidents, and framing these as a momentary loss of control) were all well-known tactics abusive men use to manage their image (Adams et al., 1995; Anderson & Umberson, 2001). Research interviews are opportunities for men, as Boonzaier (2014) puts it, to construct themselves as ‘typical men’ as opposed to ‘violent monsters’. In Harold's narrative, he also implied that a police officer, i.e., an outsider with authority, sympathetically and with allegiance (“mate”) accepted his account of what happened, i.e., that his wife drove him to his actions, and offered guidance (“get a divorce”). Harold used the police officer's apparent acceptance of his account as a way of making his reaction appear credible and reasonable. Despite recognising all of this, in the end, SD offered to pause the interview and make Harold a hot drink. He accepted. She then asked whether he wanted to skip the violence questions. He said ‘yes’. SD felt, on reflection, that she should have continued with the questions later, and that she had been too gentle. This was yet another example of us acting primarily as qualitative researchers and specifically as empathic listeners (Bluff, 2005; Rapport, 2005; Taylor, 2005) and highlights our feminised roles, soothing men's emotions, and helping along their talk (Grenz, 2005; Huysamen, 2016).

In terms of further intersectional considerations, Harold, a white man, had made flirtatious remarks to SD just before the baseline, as well as telling her he found a staff member at the substance service (another woman of colour) attractive, saying he “liked women with darker skin”. SD again did not challenge or call out these comments as offensive, unacceptable, and racially fetishizing. In their 2012 article, Päivinen and Holma (2012) reflect on a therapist-led perpetrator programme, during which a female therapist confronted a male participant for his flirtatious behaviour (texting her outside of the group session) as “belittling” and “offensive” (p.68). By contrast, Presser (2005)—like SD—chose not to call out the flirtatious and sexual comments her participants made: she explains that she did so in line with her qualitative methods training, e.g., not interrupting or judging, but also because was worried the participant would end their contact.

Although therapists and researchers both aim to build rapport and empathise (Dempsey et al., 2016), in some ways, the rules of what a researcher can do are more constraining than the rules of what a therapist can do in these situations: Dempsey et al. (2016) point out that the researcher role requires firmer boundaries and a level of distance. As KM's example with Greg indicates, one outcome of this distance is feeling unable to probe each thing participants say. Miller has similarly reflected that when participants used her interviews as therapeutic encounters, it caused her a sense of unease because she lacked formal therapeutic skills (Birch and Miller, 2000). The relationship between researcher and participant can also feel more vulnerable than that between therapist and client in that the success of our research is reliant on having a continued and good relationship. These findings raise the question of how far we, as researchers, should go in our endurance of discomfort in the name of the research.

### Managing the Seesawing Flow of Power

We encountered power dynamics within our research that were complex and multi-layered. In one way, we as researchers held power over men as participants, since we had the authority to ask the questions, use the resulting data, and name them as abusive (although not always directly). We took steps to reduce this power imbalance, for example, offering travel expenses as well as money or vouchers to recompense men for their time, and the usual reminders of participant rights such as stopping at any time. We also relinquished some level of control, e.g., allowing men to talk freely around questions, including during the structured interviews. For REPROVIDE's men, an added power dynamic was that some men were encouraged to attend by powerful institutions such as social services. There were also intersectional power imbalances along race and class lines, as SD's earlier example with Dylan hints at.

The interview can in fact create a sense of powerlessness in the researcher because they are dependent on the participant's cooperation and willingness to divulge information (Gailey & Prohaska, 2011; Hearn, 1998). It is worth mentioning that the end-of-study qualitative interviews in ADVANCE were short: around 20 min long. Men's reticence to talk at length may have been due to shame, stigma, and embarrassment, but may also have been a way for them to assert power. In our research, this was amplified by and connected to the power imbalance between us as women and the participants as (abusive) men—the very imbalance that gives rise to gendered violence. As other researchers have found (Boonzaier, 2014; Gailey & Prohaska, 2011; Hearn, 1998), interviews are sites in which these men can assert their dominance, and in turn, maintain and reproduce gendered patterns of power and authority. As a result, interviews can leave researchers feeling vulnerable and in some cases that their safety is at stake (Love et al., 2021). An example of this was SD's structured baseline interview with Billy. He had previously agreed to take part in this interview after his group treatment session at the substance service. However, on the day it was due, he had forgotten to tell his partner, who had been sitting outside the treatment room for over an hour waiting for the group to end. He told his partner that she would have to wait another hour while SD interviewed him, and SD felt anxious and uncomfortable knowing his partner was being made to wait. In a small, private room in the substance service where the interviews were conducted, SD explained to Billy the types of questions she would be asking. At this point, Billy looked at SD and said, “I don't like being judged”, in what SD perceived to be an aggravated and defensive tone. He also declared, “I have an anger problem”*.* He thereby immediately put himself in a position of power. Not only was Billy much bigger and likely stronger than SD, but he also constructed himself as someone who might at some point ‘lose control’ due to an ‘anger problem’ which SD perceived could be directed at her if Billy felt judged or upset by her questions. Pre-warning SD of his volatility afforded Billy the entitlement to react should she irk him further, which served to absolve him of any responsibility. SD felt especially vulnerable as it was near the end of the working day and most people in the building had left. In line with ADVANCE's safety protocol, SD made sure she could reach the room's panic alarm should she need to use it. SD felt especially cautious not to upset Billy further throughout the interview and skipped the questions that she felt might sound judgemental (e.g., about adverse childhood experiences), meaning his behaviour had a tangible impact on the data produced. These kinds of behaviours—justifying anger and abdicating responsibility—are the same tactics men use to justify and minimise IPVA with their partners (Adams et al., 1995; Anderson & Umberson, 2001). Hearn (1998) points out, “questions of safety and danger of the interviewer may parallel processes affecting victims” (p. 51), and indeed, our experiences gave us insight into how these men's partners must feel.

BL had a similar encounter during her end-of-study interview with Ash. For several weeks, he had not attended the ADVANCE intervention. Staff at the substance use treatment service told BL that social services had intervened in Ash's family in response to a situation that could have posed a risk to his wife and children. ADVANCE's facilitator said Ash was angry and blamed the service and was demonstrating this by not attending the group. BL managed to contact Ash and he agreed he would try to attend the group that week, which he did. He initially appeared polite and cordial towards BL. But his demeanour became aggressive during the interview. BL was also in a confined room away from other staff, and this again affected the data produced: BL felt threatened and so rushed through questions:

BL'S FIELDNOTE: Ash was curt with me from the interview's outset and appeared agitated, so I felt pressured to hurry through. At the end, when we both stood up, his body language became aggressive: he stood close to me and raised his voice in an angry tone and said he blamed the intervention and me for social services intervening with his family. He said he felt this was unfair as he had only wanted to help me by taking part. I calmly explained that I had no part in the decision to involve social services. His tone and body language continued to be aggressive. I changed the topic of conversation to reimbursement and signing forms and focused on escorting him out of the room. On reflection, I felt ambushed by his behaviour. Until then, he had been amicable, and I had not anticipated having to experience his aggressive behaviour first-hand. I felt he was exercising an attempt to control a situation over which he had no control (social services intervening) by exerting anger towards me: a lone woman in a confined space, where I had no choice but to hear him.

Notably, in his exchange with BL, Ash not only used intimidation but also emphasised that he had ‘helped’ her by taking part in the research, indicating that she should be grateful to him. Others (e.g., Grenz, 2005; Huysamen, 2016) have found that male research participants emphasised being ‘good men’ by ‘helping women researchers in need’. Presser (2005) interpreted such responses as chivalrous attempts to assert masculine authority. Boonzaier (2014) adds that ‘chivalry’ places the female interviewee in a position of ‘emphasised femininity’—a femininity that is organised around compliance and subordination to masculinity. Chivalry and highlighting ‘helpfulness’ were men's attempts to downplay their problematic selves (Boonzaier, 2014; Huysamen, 2016; Presser, 2005).

A less pernicious and more subtle example of chivalry, masculine authority, and positioning the researcher in terms of emphasised femininity featured in SD's end-of-study qualitative interview with Simon, where she asked him what he thought about the quantitative measures administered as part of the research:

SD: Any ways they could be improved?

Simon: Fine. Fine, Sandi, honestly. You’re a nice lady, you make it comfortable and easy, so…

SD: Oh, thank you.

Simon phrased his answer as if SD were seeking his reassurance about her capabilities rather than asking for his views on the measures. His answer, particularly the repetition (“fine, fine”), the reassuring discourse marker (“honestly”), the overfamiliar use of her name (“Sandi”), and the gendered label (“nice lady”) is another example of how men placed us in positions of emphasised femininity. Arguably, Simon's use of SD's name also deflected attention onto her and away from him and his participation in the programme: another way of exerting power. SD deferred to the male participant in these exchanges, using what could be interpreted as a “performance of emphasised femininity” (Boonzaier, 2014 p. 239), also noted by Boonzaier (2014) and Huysamen (2016) in their interviews. Specifically, she accepted his reply with a “thank you”, making it seem that an evaluation of her capabilities was indeed what she was seeking, rather than persisting with the question she actually wanted him to answer. Presser (2005) too did this, and writes “upon data analysis, to my chagrin, I found that the men were not the only ones to position me in gendered ways. I did so myself” (p. 2080). On one hand, we may have acted this way subconsciously: patriarchal society may have ingrained in us these feminised responses. On the other, we may have been consciously complying, in line with our qualitative methods training, since challenging men's behaviour could have caused men a sense of disempowerment or discomfort.

The way we managed power dynamics was dependent on how aware of them we were in the moment and how confident and safe we felt to manage them. These dynamics not only affected the data produced, but also intensified the emotional work involved in managing the interviews, specifically around attending to men's anger.

### Over-Investing in Men's Discourses of Change

Boonzaier (2014) has pointed out that researchers will bring their own expectations to research about men's engagement with perpetrator programmes. She interviewed men after they had completed a programme and found herself expecting a narrative from them in which they remorsefully admitted they had perpetrated violence. She writes that “my…investment in a discourse of change…meant that I would encourage its telling but also that I was more likely to enter into a relationship of ‘mutual respect’…and empathy with its teller” (p. 238). She continues, “[this] investment…was the emotional baggage I carried with me into the interviews, in order to assure myself that the men would not go on to perpetrate further violence against the women” (p. 239). We found evidence that we also invested in and encouraged a discourse of change and empathised with its teller. Empathy is an essential skill in qualitative research but can also present challenges (Love et al., 2021): in this case, hearing evidence of change that was not there. One example of us investing in a discourse of change again comes from HC's interview with Charles. He described an incident where his partner threw a tumbler at his head and rather than react angrily, he “got a basin and cleaned it up”:

HC: What would the old you have done? Gone ballistic?

Charles: Because I felt it actually just whiz past my head. I felt the wind from it.

HC: So, the new you is very restrained in all situations, and you’ve been tested.

Charles: Yes, you have to be.

HC's use of the terms “old you” and “new you”, and her response, “very restrained…all situations”, which did not directly follow from what Charles said, perhaps indicates an over-investment in his discourse of change. He heartily agreed with her framing of his change, (“yes, you have to be”) despite having only given one example of this level of ‘restraint.’

Our feelings of empathy and investing in change discourses also made us less able to recognise that men might have simply learnt ‘programme language’. According to McGinn et al. (2020), learning programme language, i.e., having explanations for IPVA that are very similar to those found in academic literature, is common, and “may be indicative of a low level of engagement with intervention and low levels of motivation to change” (p. 102). One example came from SD's end-of-study qualitative interview with Jacob. He stopped attending ADVANCE near the beginning of the programme, but said that the session content had led to positive changes:

Jacob: I’ve got to change my whole way of thinking, and just really go on the evidence. I might be getting paranoid, and something might be in my gut to say this and that, but does it mean that something's going on? Like she's cheating or anything like that? It's not fair for her to be continually trying to reassure me. I don't know if I’m making sense [laughs].

SD: No, no, no. It really does; 100%, It does make sense. It sounds really positive.

Jacob: It's that sense of foundation, isn't it?

SD: Yes!

Jacob: It makes you aware…It's better if you’re aware, because sometimes you might do things without realising it, and you’re not really sure why you’re doing it.

Jacob's phrasing of “really go on the evidence” reflects part of a session he attended where men were encouraged to use the ‘personal scientist’ approach, i.e., to determine the facts of a situation by asking what evidence exists for and against an assumption. Rather than questioning whether Jacob was ‘parroting’ programme language, SD responded with excitement about his progress. He was reporting these changes in the context of wider positive changes in his life: he had been abstinent from substances for over a year, was about to start a new career, and was (according to him) in a new healthier relationship. These factors led SD to become emotionally invested in his success and feel less sceptical over his reported changes. Her responses (“it sounds really positive…100%…yes”) led him to continue in his discourse of change. However, SD was crestfallen when he later said, “if a girl is horrible, I’ll retaliate.” Moreover, he said he would not be violent in his current relationship “because I’m with someone who wouldn't allow it to happen”, thereby blaming the previous victim for the abuse he inflicted. Change discourses were glimmers of hope in what could otherwise be emotionally challenging research. It was difficult to be empathic and encouraging without over-investing in men's change discourses, and when inconsistencies in their narratives revealed themselves, we were left feeling foolish, frustrated, and disappointed.

### Providing Boundaried Support When Men Were (Seemingly) in Need

The questions we asked in our baseline and follow-up interviews meant that men often talked about their own difficult experiences as well as their use of IPVA, including their experiences of abuse or neglect in childhood, struggles with severe mental ill-health, suicide ideation, and, among the men on ADVANCE, homelessness and high levels of substance use. These men were rarely receiving professional support for these wider issues, although ADVANCE men were receiving support from substance use treatment services. Although research ethics committees usually suggest that researchers signpost participants to helplines and services, researchers do not have the same ongoing duty of care to participants as, for example, clinicians do to patients. Moreover, research ethics committees only really suggest that researchers signpost participants if they become distressed due to the research—not if they are experiencing distress or hardship in general. Nonetheless, as researchers, particularly as feminist researchers who see the harms of oppression and systemic failures as central to our work, and who see ethical research practice as crucial, we felt obliged to support men in these situations. In some cases, this even involved informally advocating for them and approaching services to seek support on their behalf. Men often said that they had not told many people, or anyone, about their experiences before, and this strengthened our perceived obligation to help them. At the same time, we were very aware that men may have been positioning themselves as victims as a way of justifying or excusing their behaviour. Huysamen (2016) similarly found that when her interviewees had never told anyone they paid for sex, she felt a particular sense of empathy and tenderness towards them. In the example above, SD felt especially excited that Jacob had apparently changed his behaviour because he had disclosed to her (seemingly for the first time) that he was a victim of childhood sexual abuse. She felt a need to ‘rescue’ him (and his partners) from the ‘cycle of violence’ and was excited that ADVANCE might have had a positive effect. Huysamen (2016) felt that men saw the interviews with her as welcome and therapeutic confessionals. However, in our studies, although we told men they could skip questions without providing reasons, we worried that their disclosures were to an extent coerced through the researcher-participant power imbalance explored earlier. Given this, we felt especially compelled to go beyond the researcher remit to support them. But it was difficult to draw a boundary around a suitable level of support, especially because men's claims of victimhood can be a tactic to justify abuse. And as researchers not directly connected to the services, our attempts were sometimes frustratingly fruitless as well as emotionally draining.

## Discussion

In this article, we have provided a reflexive analysis of the key tensions we experienced in research with men who had perpetrated IPVA to female partners. These tensions stemmed from our multiple positions and roles—as researchers who draw on feminist traditions such as emphasising reflexivity, reciprocity, empowerment, and ethical practice (Cancian, 1992; DeShong, 2013; Doucet & Mauthner, 2012; Harding, 1987; Hesse-Biber, 2011; Olsen, 2011; Stanley & Wise, 2013; Taylor, 2005), as women, and as feminists. These positions and roles were sometimes at odds with each other. We used aspects of qualitative research skills both in our structured and semi-structured interviews, such as creating a non-judgemental space, maintaining rapport, and capturing men's voices (Bluff, 2005; Britten, 1995; Patton, 1987, 2015; Rapport, 2005; Taylor, 2005), but in doing so, we danced around potentially stigmatising language. Our use of euphemisms led us to over-protect those men who knew exactly what the euphemisms meant. We used reciprocity and mutual disclosure but found ourselves skirting the line between rapport and collusion. We helped along men's talk as empathic listeners but were mindful of how men might have mistaken our silences as support for their comments. When men got upset, we felt we needed to attend to their well-being, while simultaneously feeling they were exploiting our empathic approaches. As researchers, we felt unable to challenge abuser tactics or call out abuse, but as women and as feminists, this made us uncomfortable. We made these decisions in order to keep men engaged, which was important to us because engagement would strengthen our data and ability to generate evidence for a programme that could help women. Like others (Boonzaier, 2014; Gailey & Prohaska, 2011; Presser, 2005), we found that interviews were suffused with multi-layered power dynamics, in part because both parties belonged to both a dominant and a subordinate social group (woman/researcher - man/participant). While using strategies to redress the power imbalances that favoured researchers, we also experienced men's subtle and overt attempts to gain power and found that interviews were sites of patriarchal power and control. We sometimes shared oppressions with men that disempowered them, and at times over-empathised with men, investing in their discourses of change. We at times felt morally obliged to step beyond the researcher role and attempt to advocate for men who were victims of childhood abuse or systemic failings, while keeping in mind the abused partners. Swaying between positions and roles caused a sense of discordance and emotional confusion. All these tensions were amplified since in some cases we had interviewed female partners of the perpetrators, making the reality of their IPVA starker.

These tensions, and the ways we subtly steered and were steered in our research, had the potential to affect the data produced and in turn the evidence base around these perpetrator programmes. For example, our decisions not to use the term ‘domestic violence’ in the initial stages of ADVANCE meant we may have missed out on having frank discussions with those men who knew what our programmes were about and felt ready to hear the term. Moreover, by not challenging men, we lost the opportunity to identify and explore contradictions, minimisations, and justifications for violence (Hydén, 2014). This loss of opportunity was particularly relevant when men were allocated to the control group or dropped out of the programmes (since for men who completed the programmes, programme facilitators would have fulfilled this role). Men's displays of power led us to sometimes rush through and skip entire sets of questions. Their claims of change, and our emotional investments in change, led us to encourage these narratives in qualitative interviews. Love et al. (2021) similarly found that researchers’ emotional investments affected their interpretation of data: one researcher “felt sympathy for [a] man's adverse and violent childhood” and felt he was a victim of his female partner's behaviour, whereas the researcher who interviewed his partner was “moved by the [woman's] distress [and] timid demeanour” (p. 773). The emotional investment made it difficult to agree upon an interpretation of the relationship.

The most demanding tension for us to resolve was deciding whether to call out men's abusive behaviours. Unlike the others, this tension posed an obvious moral dilemma: not challenging men might leave them feeling vindicated around the IPVA they had inflicted. Taken to its extreme, this might exacerbate their violence and abuse or leave women feeling that researchers have absolved their partners of wrongdoing. Given this, Boonzaier (2014) questions whether the widely taught and widely adopted model of ‘good interviewing’ (empathic engagement, building rapport, not disagreeing, etc) is always appropriate. DeShong (2013) writes that she tried to probe and challenge men and raise their awareness of gender and power, such as by asking why they did not use non-violent conflict-resolution tactics and whether they felt responsible for perpetrating violence. However, she concluded that it was “virtually unattainable [to achieve meaningful consciousness raising] in a single sitting” (DeShong, 2013, p.4). Another way forward might be to train researchers on alternative interview methods. Hydén's ‘teller-focused interview’, developed for IPVA research, emphasises the facilitation of interviewees’ narratives. To our knowledge, Hydén (2014) is the only IPVA researcher who has reported successfully challenging men in interviews—in one example, on a man's use of the ‘rhetoric of exculpation’ tactic, where he claimed he hurt his wife in anger-induced blackouts. Hydén was using longitudinal interviews: she had built up a relationship with the man and cultivated a space in which she felt confident and safe to challenge him. Moreover, she had developed specific styles of questioning within the ‘teller-focused’ approach to encourage interviewees to expand on their reasoning. Her challenge led to remarkable data, with the man contradicting himself and making clear he was previously lying. However, such an approach would be difficult to incorporate into RCT research where the intention is to determine the changes that an intervention, not the interview, elicits.

In the examples presented, we often identified issues and problems in what we said and did ‘in the moment’. Qualitative researchers (e.g., Reid et al., 2018; Shaw 2010;) have previously made similar observations from reflexive analyses of their own interviews, noting times at which they did not respond ethically or appropriately, for example, by not managing power dynamics that disfavoured participants. Like Wiant Cummins and Brannon (2022), through doing this work, we have learnt, “how much we realize after-the-fact” and “how ‘easy’ it is to be reflexive with the benefit of time, distance, and hindsight” (p.94). Acting reflexively in the moment was challenging: our social positions sometimes led us to react automatically, rather than respond thoughtfully.

Despite all these complexities, we can make practical recommendations for how to conduct interview-based, and in particular reflexive and qualitative, research with men who perpetrate violence against women. We hope that these recommendations can help to prepare researchers, particularly women, for conducting interviews with violent men.

### Recommendations for Practice and Research

1. All research projects should have safety protocols in place in case perpetrators express intent to harm themselves or anyone else, including protocols for information sharing with relevant agencies and services.
2. Researchers should initially be open to using language flexibly, for example using euphemisms for ‘domestic violence’ if it means engaging men who might otherwise be deterred but should discuss how well this is working with colleagues and other researchers and how well it sits with the processes for identifying abusive men.
3. Specifically on the rapport-collusion tension, Iwi and Newman (2015) (whose recommendations for practitioners are relevant here) recommend finding a neutral stance between a collusive and an accusatory one. In practice, this means empathising when the man feels bad about his abuse and refraining from overfriendliness or opposition—instead, building a connection with the side of him that wants to change.
4. Following DeShong (2013), researchers should use probes rather than overt challenges to encourage men to talk about their justifications and reasons for their violence and abuse, such as asking men about feelings of responsibility for their behaviours and why they used abuse and violence over alternatives—keeping in mind the potential for such interjections to interfere with any intervention whose effect on behaviour change is being explored.
5. All research projects with perpetrators ought to involve space for reflection and thinking around the sorts of issues we have raised. Like Gadd (2004), we recommend reflexive analysis of all interviews, especially those that feel like they have gone wrong in some way, so that researchers can identify their skills and influences on the interview. Researchers should consider keeping reflective diaries and making fieldnotes to enable reflexivity on broader aspects of the research, not just the transcribed qualitative interview. Diaries and notes can capture specific issues such as how different uses of language and probes seem to have affected men and the data produced, researcher-participant dynamics, and the researcher's feeling of confidence and safety, which can then inform choices such as about what language to use and how to conduct interviews. Journal editors should encourage and set aside wordage and pages for authors to publish reflexive notes. As Wiant Cummins and Brannon (2022) write, presenting “raw data…allow[s] readers a chance to see how we were processing in the moment rather than with the polished shine of analysis only” (p.87): other people's reflexive analyses can be a learning opportunity for future researchers.
6. All teams should provide the opportunity for debriefs and clinical supervision, with discussion around gender dynamics in research (Gailey & Prohaska, 2011; Tyagi, 2006).
7. All teams should also ensure that researchers of all genders become sensitised to gender dynamics through reflective team meetings and training, so that they can be aware of dynamics playing out in their research and support colleagues to manage difficult dynamics.
8. Researchers working in this area should build up a scholarship around feminist methodology and interview methods in perpetrator research, e.g., via peer-reviewed articles, with support from journals (e.g., special issues), public-facing articles, and talks.
9. Finally, we echo a suggestion by Tyagi (2006) that researchers should develop peer networks for brainstorming, strategizing, and mentoring newer researchers.
